# Targeting actin inhibits repair of doxorubicin-induced DNA damage: a novel therapeutic approach for combination therapy

**DOI:** 10.1038/s41419-019-1546-9

**Published:** 2019-04-03

**Authors:** Lisa Pfitzer, Christina Moser, Florian Gegenfurtner, Anja Arner, Florian Foerster, Carina Atzberger, Themistoklis Zisis, Rebekka Kubisch-Dohmen, Johanna Busse, Rebecca Smith, Gyula Timinszky, Olga V. Kalinina, Rolf Müller, Ernst Wagner, Angelika M. Vollmar, Stefan Zahler

**Affiliations:** 1 Department of Pharmacy, Pharmaceutical Biology, Ludwig Maximilian University Munich, Munich, Germany; 2 Department of Pharmacy, Pharmaceutical Biology and Biotechnology—Biotechnology and Nanomedicine, Ludwig Maximilian University Munich, Munich, Germany; 3 Department of Physiological Chemistry, Ludwig Maximilian University, Munich, Germany; 4 CNRS, Structure fédérative de recherche Biosit, IGDR (Institut de génétique et développement de Rennes)—UMR 6290, Univ Rennes, 35000 Rennes, France; 5 MTA SZBK Lendület DNA Damage and Nuclear Dynamics Research Group, Biological Research Center of the Hungarian Academy of Sciences, Szeged, Hungary; 6 Computational Biology and Applied Algorithmics, Max Planck Institute for Informatics, Saarbrücken, Germany; 7 Helmholtz Institute for Pharmaceutical Research Saarland, Helmholtz Centre for Infection Research and Department of Pharmacy, Saarland University, Saarbrücken, Germany

## Abstract

Severe side effects often restrict clinical application of the widely used chemotherapeutic drug doxorubicin. In order to decrease required substance concentrations, new concepts for successful combination therapy are needed. Since doxorubicin causes DNA damage, combination with compounds that modulate DNA repair could be a promising strategy. Very recently, a role of nuclear actin for DNA damage repair has been proposed, making actin a potential target for cancer therapy in combination with DNA-damaging therapeutics. This is of special interest, since actin-binding compounds have not yet found their way into clinics. We find that low-dose combination treatment of doxorubicin with the actin polymerizer chondramide B (ChB) synergistically inhibits tumor growth in vivo. On the cellular level we demonstrate that actin binders inhibit distinctive double strand break (DSB) repair pathways. Actin manipulation impairs the recruitment of replication factor A (RPA) to the site of damage, a process crucial for homologous recombination. In addition, actin binders reduce autophosphorylation of DNA-dependent protein kinase (DNA-PK) during nonhomologous end joining. Our findings substantiate a direct involvement of actin in nuclear DSB repair pathways, and propose actin as a therapeutic target for combination therapy with DNA-damaging agents such as doxorubicin.

## Introduction

The anthracycline doxorubicin (Doxo) is widely used for therapy of a broad range of cancer types. However, like any other chemotherapy, treatment with Doxo induces unwanted side effects (e.g. cardiovascular morbidity due to both acute and delayed cardiotoxicity^1,2^). Nevertheless, Doxo is still an indispensable part of cancer therapy. To increase therapeutic effectiveness while reducing unwanted adverse effects, combination therapy concepts have to be developed.

One mode of action of Doxo is to stabilize the topoisomerase II complex during DNA replication, which induces DNA double strand breaks (DSB) and promotes apoptosis^3,4^. Cells respond to such DNA damage by starting various repair mechanisms. Targeting specific proteins involved in these DNA repair pathways (repair factors) has been suggested as a promising approach for cancer therapy. DSBs can be repaired by different repair pathways, depending on the functional context of the cell. Nonhomologous end joining (NHEJ) works independently of sequence homology and cell cycle. During NHEJ, DNA-dependent protein kinase (DNA-PK) is formed by binding of its catalytic subunit (DNA-PKcs) to a DNA-bound Ku70/80 heterodimer, leading to its activation. Activated DNA-PK phosphorylates itself and other targets involved in NHEJ^5–7^. The other DSB repair pathways depend on DNA end resection, i.e. the generation of single stranded DNA (ssDNA). Alternative end joining (alt-EJ) utilizes PARP-1-mediated annealing at short homologous DNA sequences (microhomologies)^8,9^. Homology directed repair (HDR) and single strand annealing (SSA), however, both depend on extensive end resection, as replication factor A (RPA)—a crucial initiation factor for both pathways—can only bind to ssDNA. During HDR, RPA mediates Rad51-dependent strand invasion of intact homologous regions on the sister chromatid during S-phase for accurate repair^10–12^. SSA anneals homologous repeat sequences that flank the DSB, which is facilitated by Rad52 bound to the ssDNA-RPA complexes^12–14^.

Surprisingly, sufficient nuclear levels of actin and its ability to polymerize have been suggested to be required for efficient repair of irradiation or UV-induced DNA damage^15,16^. It seems that DSBs have to be actively clustered in an actin-dependent way during the G1 phase of the cell cycle before being repaired in the G2 phase^17^. Very recently, nuclear Arp2/3 complex^18,19^ and myosins^18^ have been identified as crucial factors for actin-dependent mobility of DSBs for efficient repair by HDR. To date, these mechanisms have been investigated mainly in model systems that can be tightly controlled to allow for maximum mechanistic insight. However, the role of actin in the DNA damage repair processes in a complex and more clinically relevant setting is far from being understood. As the obvious requirement of actin during DSB repair suggests actin as an attractive target for combination therapy with DNA-damaging substances, we used two classes of actin-binding compounds: polymerizers, such as jasplakinolide (Jaspla)^20^ or chondramide B (ChB)^21^, and a depolymerizer, like latrunculin B (LB)^22^ in combination with the clinically relevant drug Doxo. Despite the fact that actin binders alone have shown antitumor characteristics in different experimental setups^23–25^, actin is not directly targeted yet for clinical application due to toxicity issues.

In this study we show that combination of subtoxic doses of actin-binding substances with classical chemotherapy is possible and leads to synergistic effects on tumor growth in vivo. We demonstrate the failure of DSB repair due to actin manipulation after Doxo-induced DNA damage, both in vitro and in vivo. On a molecular level, we describe inhibition of RPA recruitment during HDR and SSA, as well as decreased autophosphorylation of DNA-PK during NHEJ. We propose DNA repair inhibition by application of actin binders as a novel strategy for combination therapy with the DNA-damaging agent doxorubicin.

## Materials and methods

### Cell culture

MDA-MB-231 cells were purchased from CLS (Eppelheim, Germany), 4T1-luc2 (mouse) from Caliper-PerkinElmer (Alameda, CA, USA), T24 and HeLa from DSMZ (Braunschweig, Germany). U2OS reporter cells^26^ were kindly provided by Jeremy M. Stark (Beckman Research Institute of the City of Hope, USA). MDA-MB-231 and HeLa were cultured in DMEM (Dulbecco´s Modified Eagle Medium), T24 and U2OS in McCoy’s 5A medium, 4T1 cells in RPMI. Media were purchased from PAA Laboratories (Pasching, Austria). All cells were cultivated with 10% FCS (fetal calf serum) and 1% penicillin/streptomycin (Sigma-Aldrich) and were grown at 37 °C, 5% CO_2_. HeLa cells were tested for mycoplasma every fourth month. Cells were not used for more than 15 passages after thawing.

### Reagents

Jasplakinolide (Jaspla) was purchased from R&D Systems (Bio-Techne GmbH, Wiesbaden, Germany) and Latrunculin B (LB) from Sigma-Aldrich (Taufkirchen, Germany). Chondramide B (ChB) was isolated as described previously^27^. Doxorubicin hydrochloride (Doxo) was purchased from Sigma-Aldrich (Taufkirchen, Germany). Antibodies are listed in Supplementary information.

### In vivo mouse models

All animal experiments were approved by the Government of Upper Bavaria in accordance with the German Animal Welfare and Institutional guidelines.

#### Tumor growth model

5 × 10^6^ MDA-MB-231 cells were injected subcutaneously into the flank of SCID mice (C.B-17/IcrHanHsd-Prkdcscid, Harlan Laboratories, USA). Forty mice were divided equally into four treatment groups: solvent (DMSO), two single groups treated intravenously either with Doxo 1.5 mg/kg body weight (once a week) or ChB 0.6 mg/kg body weight (thrice a week) and a combination treatment group.

#### In vivo comet

1 × 10^6^ 4T1-luc cells were injected subcutaneously into BALB/cOlaHsd mice (Envigo, Netherlands) and tumors grown for 7–9 days. Mice were then treated with 3 mg/kg Doxo i.v. or in combination with 0.1 mg/kg LB i.p. for 24 h. Tumors were harvested, single-cell suspensions prepared and cells seeded accordingly for alkaline comet assay.

### Staining of tumor sections

Tumors were removed, fixed in formalin and embedded in paraffin. Sections were stained with anti-Ki67 antibody (Abcam, USA) and visualized with the Vectastain ABC Kit (Vector Laboratories, USA) according to the manufacturer’s instructions. Images were taken on an Olympus BX41 microscope (Olympus, Center Valley, PA, USA). Ki67-positive cells were counted with ImageJ (National Institute of Health (NIH), USA) and normalized to the total number of cells.

### Alkaline comet assay

For quantification of DNA strand breaks, alkaline comet assay (single-cell electrophoresis) was performed as described in the Supplementary information.

### Fluorescence correlation spectroscopy

FCS measurements were performed on a Leica TCS SP8 SMD. Cells were transfected with Actin-GFP (addgene 21948) using FuGENE® HD (Promega, Mannheim, Germany) 24 h before the FCS measurement. The effective volume (*V*_eff_) and structure parameter (*κ*) were measured before each experiment using 1 nM ATTO488 dye solution (ATTO-TEC GmbH, Siegen, Germany). For every nucleus three different points were measured (45 s per point). After the zero-time point measurement, Doxo [250 nM] was added. FCS curves were analyzed with the Picoquant SymPhoTime V 5.2.4.0 software and fitted with a single diffusing species and a triplet state.

### I-SceI-based reporter cells

FACS analysis of U2OS cells harboring I-SceI-based reporter systems was performed as described previously^26^. Briefly, U2OS cells were transfected with pCBASceI plasmid (addgene 26477) using FuGENE® HD for 6 h, and cells then treated with the indicated actin binders. DMSO treatment served as positive, untransfected cells as negative control. After 72 h cells were harvested and flow cytometry analysis of GFP-positive cells performed on a FACSCanto™ II (BD Biosciences, Heidelberg, Germany).

### Duolink® assay

After treatment of cells with the indicated substances or transfection with actin plasmids YFP-NLS-Beta-Actin (addgene 60613), YFP-NLS-Beta-Actin-G13R (addgene 60615), YFP-NLS-Beta-Actin-S14C (addgene 60614), Duolink® assay was performed according to the provider (Sigma Aldrich, Taufkirchen, Germany). The detailed procedure is described in the Supplementary information.

### Life cell imaging of actin

For life cell imaging of cells, a stage top incubator (Okolab, Pozzuoli, Italy) was installed on a Leica TCS SP8 SMD. To visualize actin cells were transfected with actin-GFP (addgene 21948), actin-mCherry (addgene 54966), or nuclear actin-Chromobody® (ChromoTek GmbH, Planegg-Martinsried, Germany) using FuGENE® HD.

### Foci formation assay

Treated cells were fixed with 4% PFA (paraformaldehyde), permeabilized with 0.2% Triton X-100, incubated in primary and secondary antibodies and costained for actin and nuclei with rhodamine phalloidin (Thermo Fisher Scientific) and Hoechst (Thermo Fisher Scientific), respectively. Foci were either counted manually or with the FindFociGUI (ImageJ) plugin.

### Chromatin association assay

Chromatin association was measured as described previously^28^. In short, HeLa cells were treated with Doxo for 2 h alone or in combination with the respective actin substance and harvested in cold PBS. Cells were resuspended in extraction buffer (0.2% Triton X-100) and incubated on ice for 5–10 min, washed and fixed with 4% PFA. Fixed cells were incubated in primary antibodies, washed once and incubated with the secondary antibodies, washed again and FACS analysis performed.

### Flow cytometry

Cell death was determined by YoPro (Thermo Fisher Scientific) exclusion assay. Treated cells were harvested and stained with 1 µM YoPro. YoPro-positive cells were detected by flow cytometry on a BD FACSCanto II Flow Cytometer. To analyze the impact of combination therapy on cell cycle arrest, 7-AAD (Thermo Fisher Scientific) was applied. Cells were plated in 12-well plates (1 × 10^5^ cells/well) and cultured for 24 h followed by treatment with Doxo and the respective actin substances. After 48 h cells were harvested and mixed with 250 µl methanol at 4 °C. Fixed cells were washed once more with PBS, resuspended and stained with 7-AAD according to the manufacturer’s instructions. In an FSC/SSC plot cell debris was excluded, then cell aggregates of two or more cells were removed and cell cycle phases were analyzed with FlowJo software (Tree star Inc., Ashland, OR, USA).

### Western blot and coimmunoprecipitation

Western blot was performed according to the previously published protocol^29^. For coimmunoprecipitation cells were lysed in hypotonic buffer, followed by nuclear lysis. Protein lysates were incubated in pulldown antibody for 2 h at 4 °C and beads (Protein A/G PLUS, Santa Cruz; normal mouse antibody (CST) and beads without antibody served as control) added for one more hour. Beads were washed with nuclear lysis buffer and SDS-PAGE performed. Buffer recipes are listed in the Supplementary information. ChemiDoc™ Touch Imaging (Bio-Rad) System was used for detection. Quantification of protein amount was done by Image Lab^TM^ Software (Bio-Rad).

### RPA-2/actin-binding structure model

Structure modeling of the RPA-2/actin protein−protein interaction was performed with HHpred, PredUs, SPPIDER, ConSurf -, ClusPro, and RosettaDock as described in detail in the Supplementary information.

### Statistics

GraphPad prism was used for statistical analysis. Error bars indicate mean values ± SEM of three independent experiments unless stated otherwise. Student’s *t* test and one-way ANOVA with Bonferroni post hoc tests were conducted as indicated in the respective figures. *p* values ≤ 0.05 were considered significant unless otherwise stated.

## Results

### Combination treatment of chondramide B and doxorubicin inhibits tumor growth in vivo

**Fig. 1:**
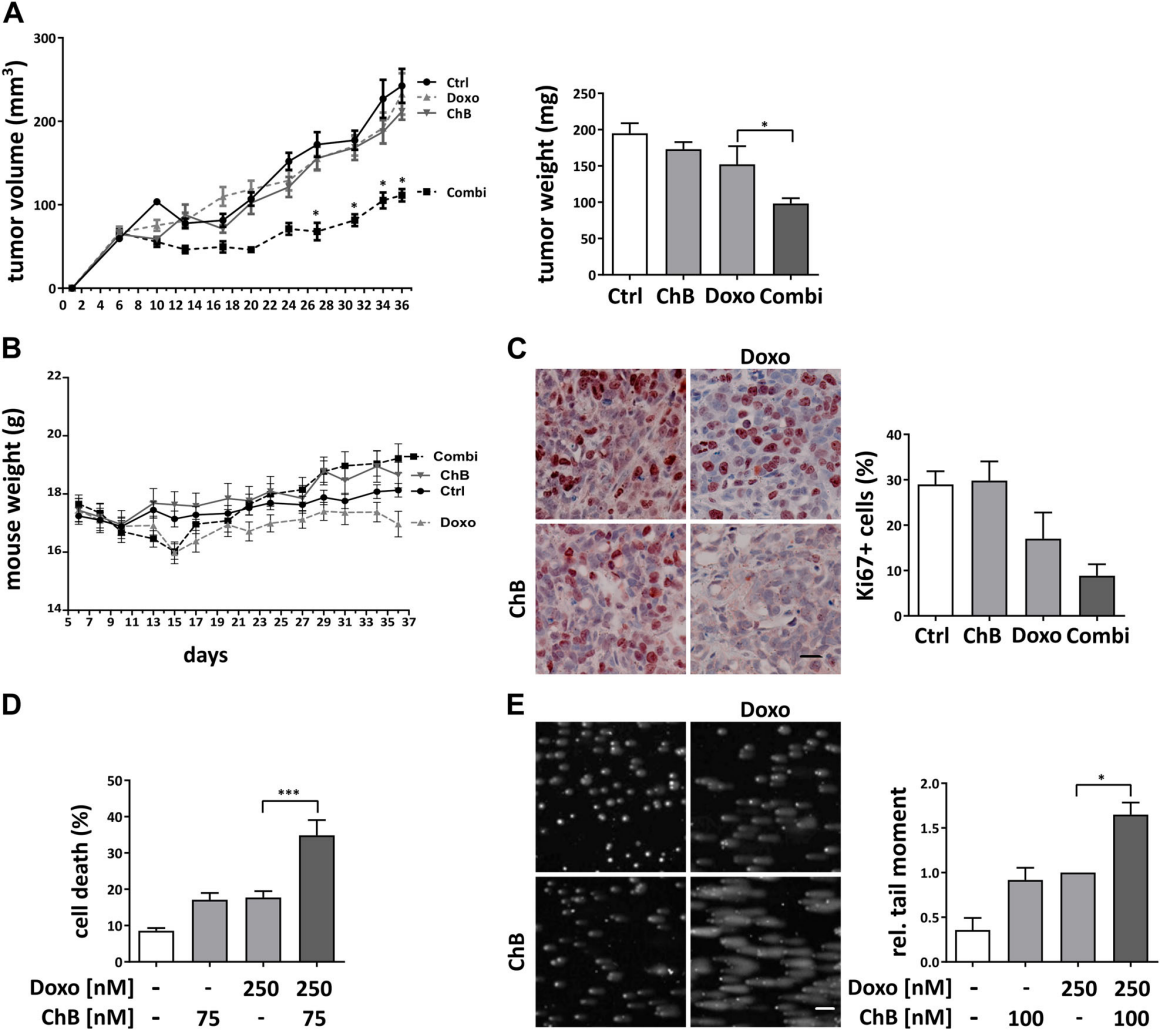
Combination treatment of Doxo and ChB inhibits tumor growth. **a−c** MDA-MB-231 cells were injected into the flank of SCID mice. Mice were treated with Doxo or ChB alone or in combination, DMSO served as control (Ctrl). Tumor size was measured at the indicated time points and tumor weight at the end of the experiment (**a**), mouse weight was observed at the indicated days (**b**). Ki67 staining was performed on paraffin-embedded tumor sections and Ki67-positive cells quantified (*n* = 4). One representative staining per treatment group is shown; ×40 magnification, scale bar 10 µm (**c**). **d** Cell death analysis was conducted with MDA-MB-231 by flow cytometry analysis of YoPro-positive cells in triplicates (*n* = 3). **e** T24 cells were treated with the indicated substances for 3 h and alkaline comet assay performed (*n* = 3). **a** (left), **b** two-way ANOVA, *p < 0.001. **a** (right), **c**, **d**, **e** one-way ANOVA, *p < 0.05, ***p < 0.005. Scale bar 5 µm

The feasibility of using an actin polymerizer in cancer treatment as a new therapeutic approach for combination chemotherapy was evaluated in a xenograft mouse model. For this purpose chondramide B (ChB) was used, since we already had successfully used this compound in previous in vivo studies^30^. Treatment regimes were established that do not show an influence of single treatments on tumor growth (Fig. 1a). In contrast, MDA-MB-231-bearing SCID mice showed significantly reduced tumor volume after treatment with ChB and doxorubicin (Doxo) in combination (Fig. 1a). The combination therapy was well tolerated in vivo, indicated by the unaltered body weight of treated mice (Fig. 1b). The tumor tissue was examined for the proliferation marker Ki67 and in tendency showed reduced proliferation rates (Fig. 1c), however, without reaching statistical significance. In vitro, MDA-MB-231 cells showed a synergistic cell death induction by low-dose combination treatment (Fig. 1d). To analyze, whether the enhanced cell death observed in MDA-MB-231 cells after combination treatment is related to elevated DNA damage levels, a comet assay was performed. Low-dose combination therapy led to increased DNA DSB levels in cells compared to Doxo treatment alone (Fig. 1e), suggesting an influence of ChB on Doxo-induced DNA damage.

### Actin manipulation inhibits distinctive DSB repair pathways

**Fig. 2:**
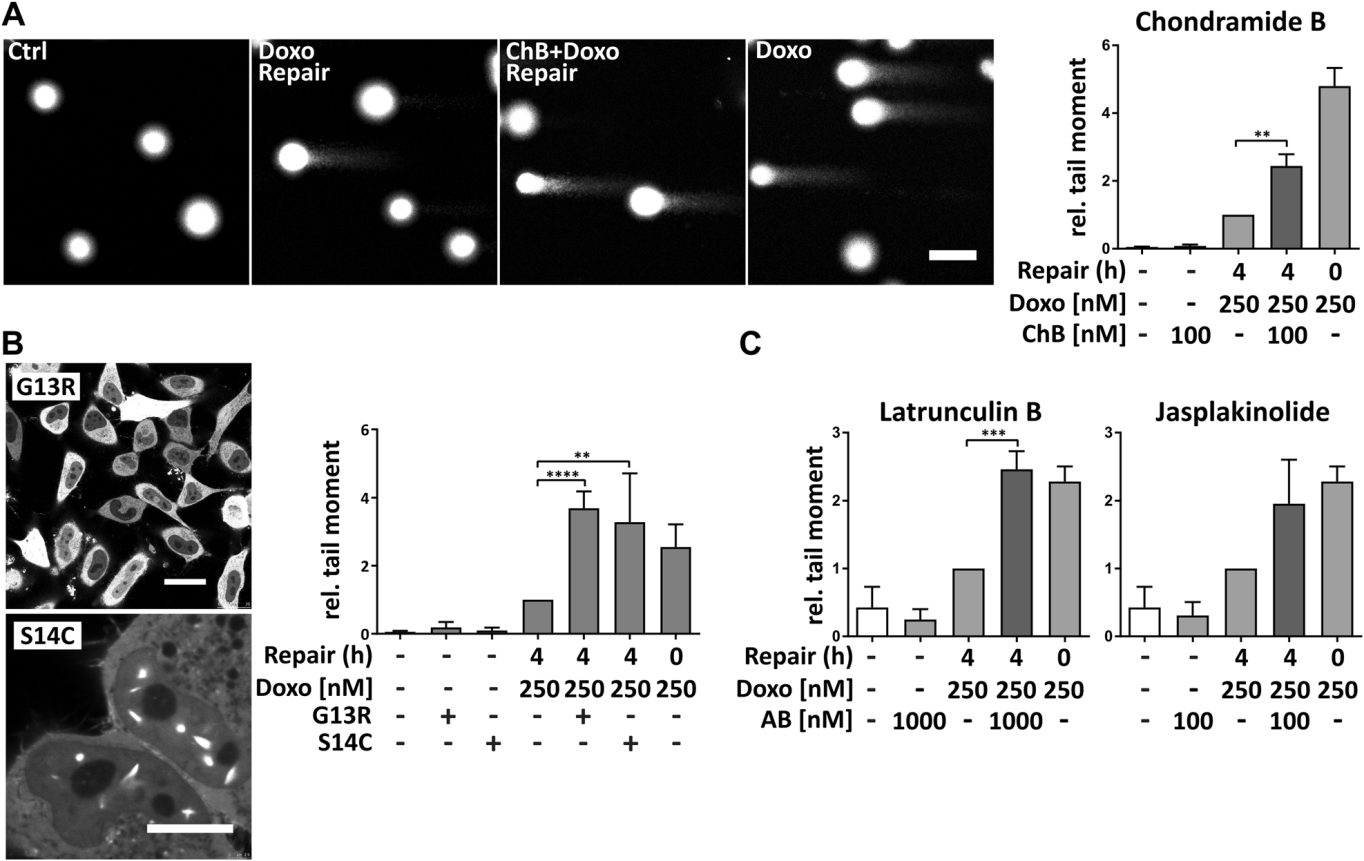
Actin manipulation inhibits repair of Doxo-induced DNA damage. **a−c** HeLa cells were treated with Doxo for 2 h, medium removed and cells incubated in DMEM (repair time). Treatment without repair served as positive control, DMSO treatment as negative control. Alkaline comet assay was performed with cells treated additionally with the indicated actin substances (**a**, **c**), or transfected with the indicated actin mutant plasmids (**b**). Images were analyzed with OpenComet, ImageJ and relative tail moments calculated. Values normalized on Doxo repair samples, mean values of at least three independent experiments are depicted. **a**, **b**, **c** one-way ANOVA, **p < 0.01, ***p < 0.005, ****p < 0.0001. **a** scale bar 5 µm, **b** upper panel scale bar 7 µm, lower panel scale bar 3 µm

To investigate, whether the higher level of damaged DNA after combination treatment is caused by additional DNA damage induction, or an inhibition of DNA DSB repair by ChB, the comet assay was performed with a different set-up. Cells were treated with ChB or Doxo alone or with the combination, followed by 4 h of repair time (i.e. without Doxo) with or without additional ChB. Treatment with the actin polymerizer ChB alone did not induce DNA damage (Fig. 2a). However, addition of ChB to the medium only during repair time increased DNA damage levels (Fig. 2a). Thus, ChB impaired the repair of Doxo-induced DNA damage. To examine whether the observed reduced repair capacity can be attributed to changes in actin states, we overexpressed actin mutants. Actin mutant plasmids can be used as a tool to either increase the G-actin pool (by using the polymerization defective mutantYFP-NLS-Beta-Actin-G13R) or to increase the F-actin pool (by expressing the hyperpolymerizing mutant YFP-NLS-Beta-Actin-S14C) in both cytoplasm and nucleus of a cell. Interestingly, repair of Doxo-induced DSBs was inhibited irrespective of whether polymerization was inhibited or induced (Fig. 2b). This finding could be verified by application of the classical actin binders jasplakinolide (Jaspla), an actin polymerizer, and the depolymerizer latrunculin B (LB), which both inhibited successful repair of Doxo-induced DSBs (Fig. 2c). The use of two further structurally unrelated compounds (miuraenamide A, an actin polymerizer, and Chivosazole A, an actin depolymerizer) showed similar effects (Supplementary Fig. S1). Again, no increased DNA damage could be detected with any actin compound alone (Fig. 2b, c and Fig. S1). Hence, enhanced DSB levels after combination treatment cannot derive from accumulation of DNA damage by single compound treatment, but are rather caused by defects in DNA damage repair due to actin manipulation.

**Fig. 3:**
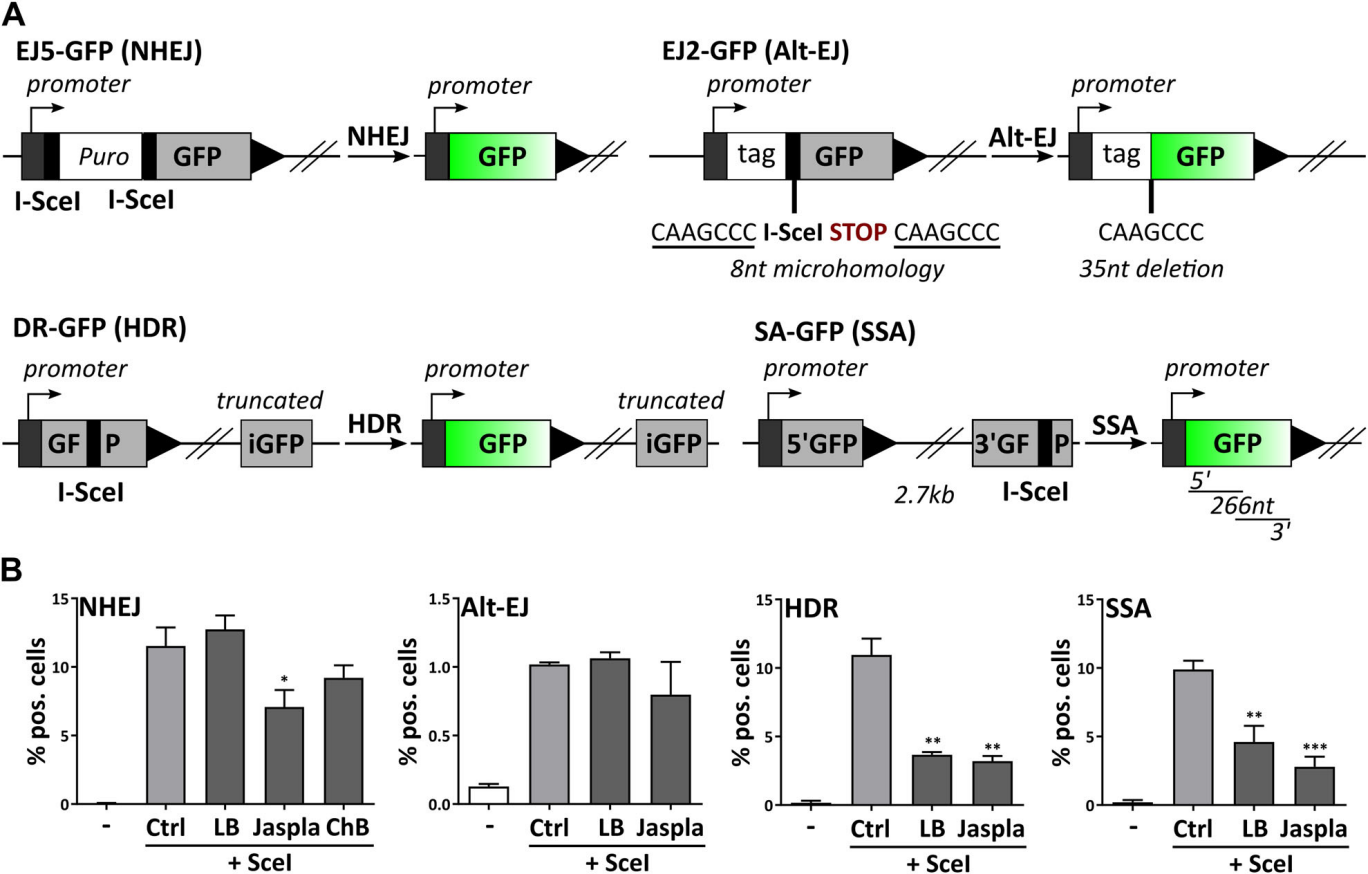
Actin-binding substances inhibit distinctive DSB repair pathways. **a** Schematics of all used reporter cell lines. **b** U2OS cells expressing one of each reporter system were transfected with pcBASE and then cultivated with or without the indicated actin substances (LB [500 nM], Jaspla [50 nM], ChB [75 nM]) for 72 h. Percentages of GFP-positive cells were measured by flow cytometry. Experiments performed in duplicates, mean values of three independent experiments are shown, one-way ANOVA, *p < 0.05, **p < 0.01, ***p < 0.005

We used I-SceI-based reporter cell lines to study DSB repair capacity of the four most important DSB repair pathways NHEJ, alt-EJ, HDR, and SSA^26^ (Fig. 3a). All I-SceI reporter cell lines express GFP-expression cassettes that are interrupted by one or more recognition sites for the endonuclease I-SceI. Transient overexpression of I-SceI leads to one or more cuts in the GFP cassette. Successful repair via the respective DNA repair pathway restores the correct GFP sequence, resulting in expression of functional GFP. Treatment with actin binders after induction of DSBs by I-SceI overexpression led to differential responses. Actin polymerization by Jaspla and ChB inhibited NHEJ, whereas LB did not reduce NHEJ efficiency (Fig. 3b). Alt-EJ was not influenced by any of the applied actin-binding substances (Fig. 3b). In contrast, both homology-dependent pathways, HDR and SSA, were surprisingly impaired independently of the class of applied actin binders (Fig. 3b). Manipulation of actin led to inhibition of distinctive DSB repair pathways, suggesting a multifaceted role of actin during DNA repair.

### The state of nuclear actin is altered by doxorubicin

**Fig. 4:**
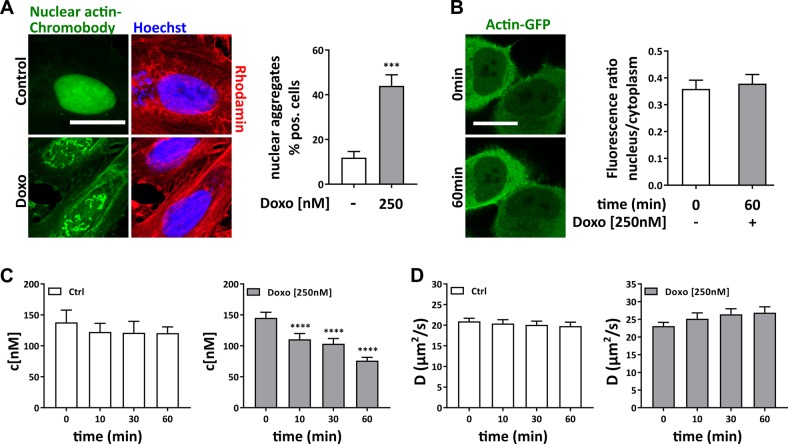
Doxo-induced DNA damage affects nuclear actin state. **a** HeLa cells were transfected with nuclear actin-Chromobody® and treated with Doxo for 2 h. Cells positive for nuclear actin aggregates were counted. Mean values (*n* = 3) are depicted, scale bar 5 µm. **b** Images of actin-GFP overexpressing HeLa cells were taken before and 1 h after addition of Doxo and fluorescence intensity measured, scale bar 5 µm. Graph shows ratio of measured intensities of nucleus to cytoplasm of up to 20 cells. **c**, **d** Cells overexpressing actin-GFP were treated with Doxo and single points FCS measurements of nuclear actin were performed. Nuclear concentrations (panel **c**) of actin (c[nM)] and diffusion coefficients (panel **d**) of nuclear actin (D(µm^2^/s)) were determined in three independent experiments (up to 10 cells per experiment). **a**, **b** unpaired *t* test, two-tailed, ***p < 0.005. **c**, **d** one-way ANOVA, ***p < 0.005, ****p < 0.0001

To evaluate if the inhibition of DNA repair is accompanied by a change in actin dynamics in the nucleus, we analyzed the state of nuclear actin before and after Doxo treatment. HeLa cells expressing the nuclear actin-Chromobody® (actin antibody fused to TagGFP and NLS) showed pronounced nuclear actin aggregates upon Doxo treatment (Fig. 4a, principle published in ref. ^16^), which seem not to be identical to F-actin, since they are not labeled by rhodamine-phalloidin (Fig. 4a). We performed fluorescence correlation spectroscopy (FCS) to measure concentration and diffusion of free (i.e. mobile) actin in the nucleus. Concentrations of free actin-GFP in the nucleus were significantly decreased in a time-dependent manner upon Doxo-mediated DSB induction whereas diffusion coefficients remained at a similar value (Fig. 4b and Supplementary Fig. S2). This indicates a transition of nuclear actin from a mobile to an immobile (bound or polymerized) state, since a nuclear export of actin-GFP upon DSB induction was not detected in an independent experiment based on fluorescence intensity measurements (Fig. 4c). We therefore hypothesize that the state of nuclear actin is functionally important for DNA damage-induced processes, and changes upon treatment with Doxo.

### Actin polymerization or depolymerization inhibits recruitment of nuclear DNA damage repair factors

Due to the observed reorganization of nuclear actin upon DNA damage induction, and the effect of actin manipulation on specific DNA repair pathways, we examined whether nuclear actin directly interacts with specific DNA repair proteins.

**Fig. 5:**
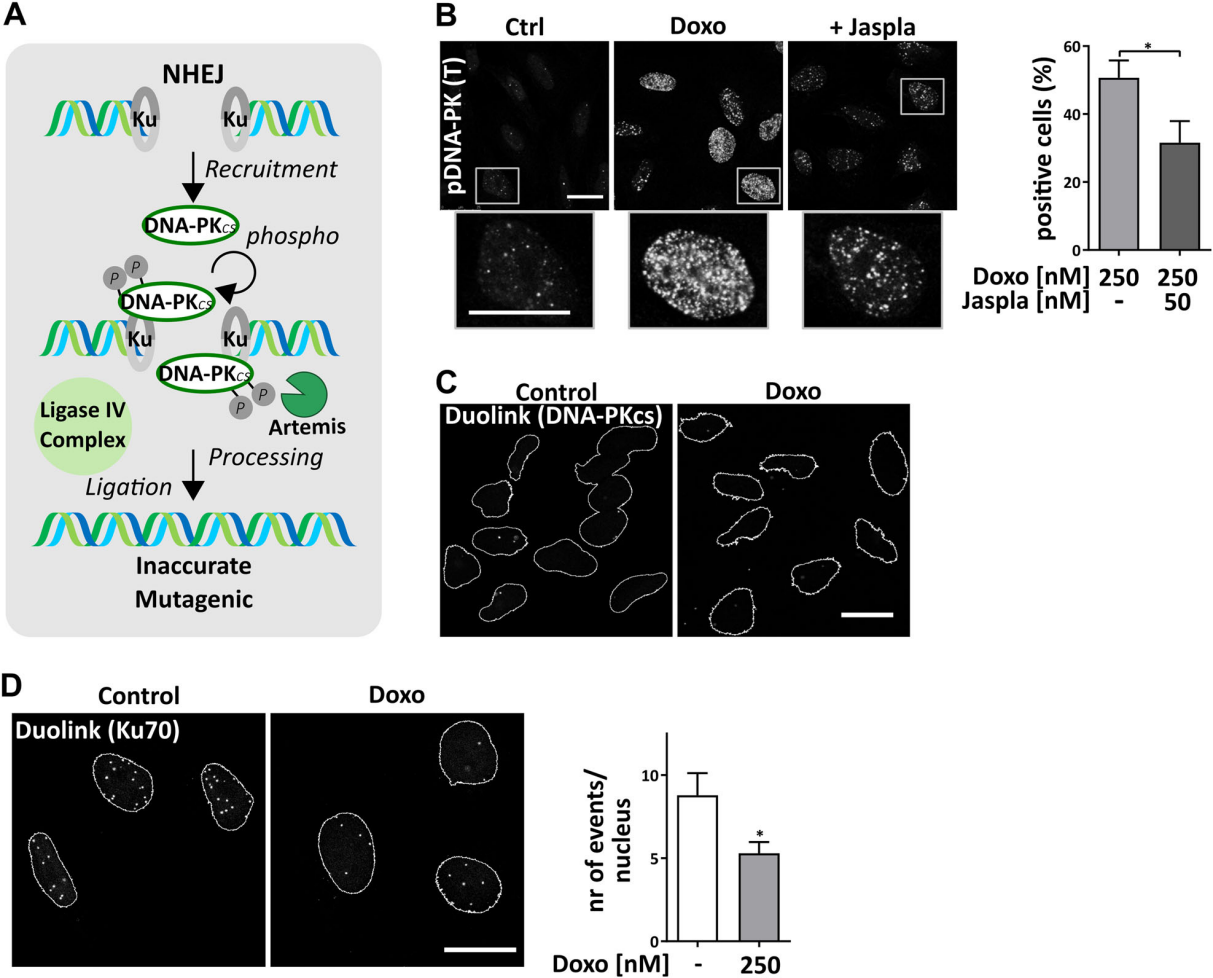
Actin binders reduce DNA-PK autophosphorylation. **a** Simplified flow chart of NHEJ. **b** Numbers of p-DNA-PK (T2609) foci were counted and cells with at least 70 foci/nucleus defined as positive cells, scale bars: 5 µm. **c**−**e** Duolink assay was performed according to the manufacturer’s suggestions. Antibodies against actin and DNA-PKcs were used for Duolink assay (nuclear area is shown and nuclear outlines depicted in white) (**c**). **d** Antibodies against actin and Ku70 were applied after treatment of cells with Doxo. Positive events were counted for each nucleus for at least three independent experiments. **b**, **d** unpaired *t* test, two-tailed, *p < 0.05. NHEJ nonhomologous end joining

NHEJ was inhibited by the actin-binding substances Jaspla and ChB (Fig. 3b). The major key player for NHEJ is DNA-PK, which is formed by binding of its catalytic subunit DNA-PKcs to DNA-bound Ku70/80, leading to its autophosphorylation and activation (Fig. 5a). Upon Doxo treatment, foci formation of autophosphorylated DNA-PK was significantly reduced in cells treated with Jaspla (Fig. 5b). A proximity ligation assay (Duolink®) showed that nuclear actin is not directly bound to DNA-PKcs under resting conditions or after stimulation with Doxo (Fig. 5c), but to the respective recruitment protein Ku70 under control conditions (Fig. 5d). This binding was reduced upon DSB induction (Fig. 5d). Nuclear actin is therefore directly associated with a subunit of DNA-PK and, as a consequence, manipulation of actin might lead to decreased DNA-PK activation. However, we were not able to consistently show the interaction of actin and Ku70 in a Co-IP approach (data not shown).

**Fig. 6:**
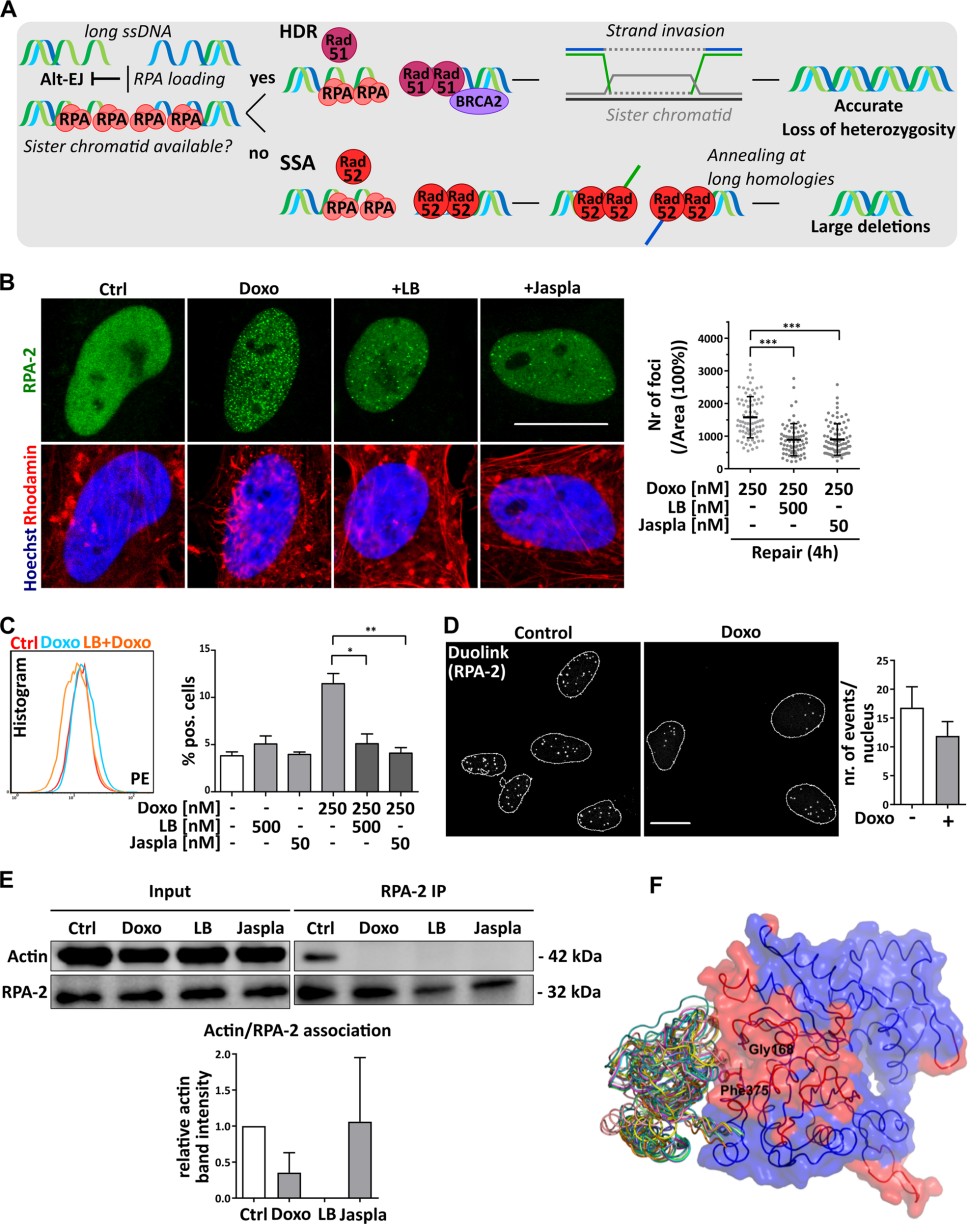
Recruitment of RPA-2 is inhibited upon actin manipulation. **a** Simplified flow chart of RPA-mediated repair. **b** HeLa cells were treated with the indicated substances for 2 h, followed by incubation in DMEM ± actin substances (repair). RPA foci were visualized by antibody staining and foci of positive cells counted and normalized on nuclear area. At least 25 nuclei were analyzed for each sample (error bars ± SD, *n* = 3), scale bar 5 µm. **c** Upon treatment, free RPA was extracted with extraction buffer, cells fixed with 4% PFA and stained for RPA-2 and flow cytometry performed (performed in duplicates, *n* = 3). **d** Duolink assay was performed with antibodies against actin and RPA-2 after treatment of cells with Doxo (nuclear area is shown and outlines depicted in white, scale bar 5 µm). Positive events were counted for each nucleus (*n* = 3). In total, at least 250 cells were analyzed for each treatment condition. **e** Nuclear extracts were prepared from treated HeLa cells, immunoprecipitated with RPA-2 antibody and immunoblotted for actin and RPA-2. Band densities were quantified and calculated as a ratio of actin intensity to RPA-2. Graph shows mean values (*n* = 3) normalized on control. One exemplary blot is shown on the left. **f** Ten best-scoring docking poses for the RPA-2/actin complex. All proteins are shown in ribbon representation. Additionally, semi-transparent surface of actin is shown, colored red and blue depending on whether a residue has been predicted to lie on a protein−protein interaction interface by SPPIDER or not. Gly168 and Phe375 are shown in stick model. **b**, **c**, **e** one-way ANOVA, *p < 0.05, **p < 0.01, ***p < 0.005. **d** unpaired *t* test, two-tailed, *p < 0.05

Interestingly, both HDR and SSA were affected by actin-binding substances, whereas alt-EJ was not (Fig. 3b). Hence, effects on key mediators that are shared by HDR and SSA can be the cause for differential responses to actin manipulation. Replication factor A (RPA) is composed of three subunits (RPA-1/2/3) and its recruitment to ssDNA is required for the initiation of both HDR and SSA and for the inhibition of alt-EJ (Fig. 6a). Recruitment of RPA-2 after Doxo-mediated damage induction was diminished by LB and Jaspla, as demonstrated by the presence of fewer RPA-2 foci in the nuclei (Fig. 6b), and a decreased association of RPA-2 and chromatin (Fig. 6c). RPA-2 was observed to be bound to nuclear actin under control conditions and the binding was in tendency (but not significantly) decreased upon DSB induction, as shown in a proximity ligation assay and coimmunoprecipitation (Fig. 6d, e), indicating a release of RPA-2 from actin upon DNA damage. We performed structural modeling of G-actin and consistently identified a conserved site predicted to be part of a protein−protein interaction interface in the vicinity of the residues Gly168 and Phe375. Global docking with subsequent local refinement offers several potential conformations for the RPA-2/actin complex, where RPA-2 lies on the predicted protein−protein interaction interface (Fig. 6f). In conclusion, we could show that nuclear actin is directly involved in the recruitment of the DNA repair factor RPA and that actin manipulation impairs RPA recruitment to the site of DNA damage, resulting in an inhibition of the RPA-dependent repair pathways HDR and SSA. Since repair pathways are also linked to the cell cycle state, we investigated cell cycle alterations by our treatment regimes. Neither LB nor Jaspla caused any cell cycle arrest at the concentrations used. Doxo itself caused a G2 arrest, which was, however, not altered by the combination treatment (Supplementary Fig. S3, left panel). Overall transcription was transiently reduced after Doxo treatment, but recovered during the repair phase. This process was not altered in the presence of either Jaspla or LB (Supplementary Fig. S3, right panel). General events essential for DNA repair, like the activation of ATM-Chk2 pathway and local chromatin relaxation, were not altered by actin targeting (Supplementary Fig. S4).

In order to investigate whether the observed effects are limited to Doxo-induced DNA damage, we also tested etoposide, cisplatin and irradiation in combination with the actin-binding compounds. Etoposide-induced DNA damage was very rapidly repaired in a complete manner, irrespective of whether the actin compounds were present or not (Supplementary Fig. S5). Levels of topoisomerase II were also not changed by chondramide, jasplakinolide, latrunculin B, Doxo or cisplatin (Supplementary Fig. S6), indicating that topoisomerase is not the central player in this context. DNA damage by cisplatin was quickly repaired by HeLa cells. Latrunculin B significantly inhibited repair, while chondramide B or jasplakinolide only showed a tendency to do so (Supplementary Fig. S7, upper panel). However, formation of RPA-2 foci after treatment with cisplatin was significantly inhibited by both latrunculin B and jasplakinolide (Supplementary Fig. S7, lower panel). Finally, irradiation with 2 Gy also caused a DNA damage, which was quickly repaired. Chondramide B, jasplakinolide and latrunculin B all significantly inhibited DNA repair (Supplementary Fig. S8, upper panel) and recruitment of RPA-2 (Supplementary Fig. S8, lower panel)

### Latrunculin B impairs repair of doxorubicin induced DSBs in vivo

**Fig. 7:**
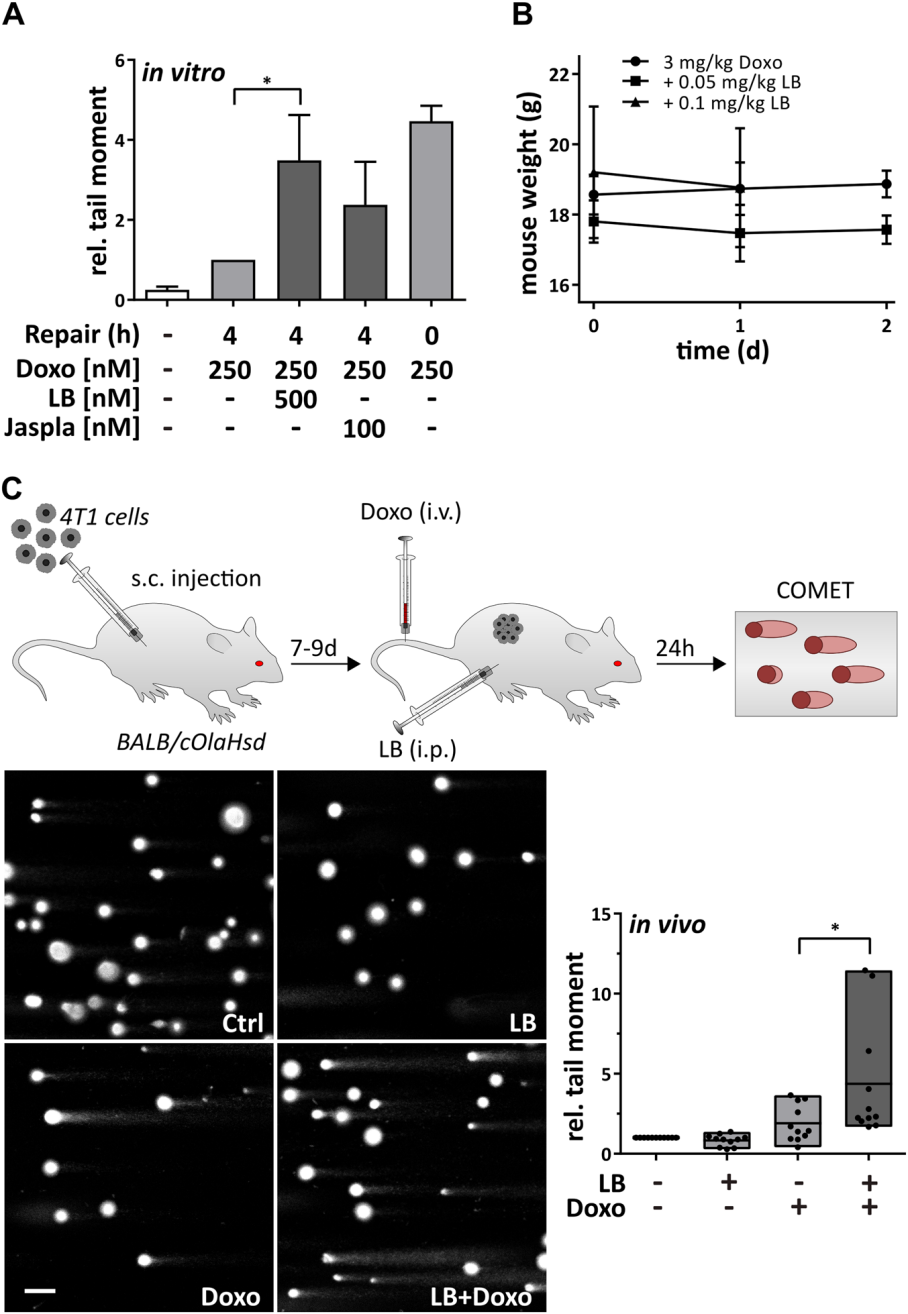
LB inhibits DNA repair in vivo. **a** Alkaline comet assay was performed in 4T1 cells as described above. **b** Mice were treated i.v. with Doxo in combination with the indicated LB concentrations (i.p.) and mouse condition and weight observed for 48 h (two mice per condition). **c** Mice were injected with 1 × 10^6^ 4T1 cells (s.c.) and tumors grown up to 9 days. Mice were then treated with the indicated substances for 24 h, tumor cells isolated, and alkaline comet assay performed. Exemplary images are shown (scale bar 5 µm), values normalized on untreated mice (*n* = 11), *p < 0.05, one-way ANOVA, Sidak’s multiple comparisons test

To assess whether repair of Doxo-induced DSBs can be inhibited by additional treatment with actin binders also in vivo, a murine tumor model was established with 4T1 cells (murine breast cancer). In vitro, the comet assay revealed a stronger inhibition of repair in 4T1 cells by LB than Jaspla (Fig. 7a), and demonstrates that the observed effect also occurs in murine cells. Since LB was tolerated well in mice if injected i.p. (Fig. 7b), an in vivo experiment was performed with Doxo treatment of mice alone or in combination with LB, and the extent of DNA damage was determined in isolated tumor cells by comet assay. LB alone did not show any effect, but in addition to the chemotherapeutic Doxo led to a strong increase of damaged DNA at subtoxic, tolerable concentrations (Fig. 7c). To conclude, the impairment of DNA repair factor recruitment by application of actin-binding substances leads to an enhancement of DNA damage levels induced by chemotherapy in vivo and provides a new approach for combination therapy.

## Discussion

The existence of nuclear polymerized actin and its function have been a matter of debate for many years in cell biology^31^. Very recently it has turned out that actin is involved in the repair of damaged DNA^16–19^. Actin is currently hypothesized to be the substrate for clustering and relocalization of DNA breaks during HDR^18,19^. The aim of our study was to investigate whether this mechanism can be modulated by actin-binding compounds, and whether such compounds might be feasible for combination therapy with DNA-damaging drugs like Doxo.

Although latrunculin A has been reported to inhibit yH2AX foci formation^32^, we did not observe the same effect with LB or Jaspla at the low concentrations we used (Supplementary Fig. S4). Therefore, general obstruction of DNA damage seems not to be the cause of impaired DNA repair upon targeting of actin. Also cell cycle status or overall transcription are not influenced by the low concentrations of actin binders we used (Fig. S3). Consequently, actin seems to directly influence specific DNA repair factors. When using reporter cell lines as an unbiased approach to detect changes in various repair pathways due to the use of actin-binding compounds, we made two surprising observations: First, not only the homology-dependent pathways HDR and SSA were affected, as was to be expected from previous work^18,19^, but also NHEJ. This is of importance, since in cancer cells, DSBs induced by Doxo are mainly repaired by NHEJ, which thus represents a promising target for combination chemotherapy. Furthermore, NHEJ is an attractive target, since such an error-prone repair pathway will likely lead to therapy-induced malignancies^33,34^. In this case, only actin polymerizers (ChB and Jaspla) had an effect, in contrast to latrunculin B (a depolymerizer). With our proximity ligation assays we show that actin directly interacts with Ku70 in the nucleus, but not with the catalytic subunit of DNA-PK. Recruitment of the Ku70/80 heterodimer to the DSB is critical for the formation and activation of DNA-PK. Actin manipulation significantly decreased autophosphorylation (i.e. activation) of DNA-PK, highlighting the functional importance of the interaction of actin with the DNA-PK subunit Ku70. Andrin et al. have previously demonstrated that Ku binds to F-actin and that actin manipulation leads to perturbed retention of Ku80 at the DNA break^15^. The obvious reduction of direct Ku7−actin interaction upon damage induction we describe here might be due to a change of conformation of the actin polymers after Doxo treatment: these seem not to be regular F-actin filaments (no phalloidin binding), and might no longer be able to support binding of Ku70. Interestingly, we have recently shown that treatment of cells with an actin polymerizing compound also elicits polymeric actin aggregates, which, in contrast to F-actin filaments, are no longer able to bind AMOT, a regulator of the YAP signaling pathway^35^. The drop in mobile nuclear G-actin levels after Doxo without any signs of nuclear export support the notion of actin aggregation upon DNA damage.

**Fig. 8:**
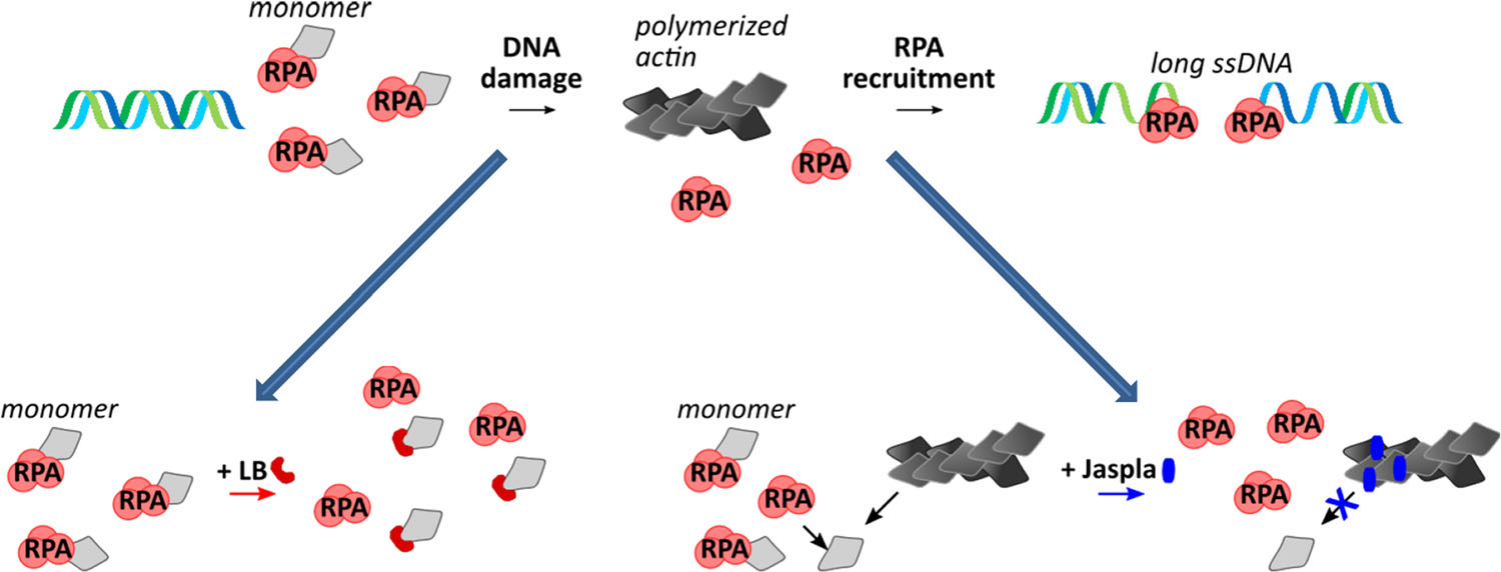
Schematic model of the interaction of RPA-2 with nuclear actin and its alteration by actin depolymerizing or polymerizing compounds

The second important finding is that HDR and SSA are inhibited by both manipulations that cause either actin polymerization or depolymerization. This observation is at first view puzzling. However, it can be readily reconciled with the recent findings that F-actin is mandatory for HDR^18,19^: depolymerization by LB inhibits F-actin formation, and, thus might hinder HDR, while Jasp causes formation of actin aggregates, which are not functional for DSB clustering, and, consequently could also inhibit HDR. It has also been previously reported that treatment with Jasp causes a decrease of nuclear actin dynamics as such^36^. When we had a closer look at RPA-2, a protein which is of central importance to HDR and SSA alike, we found that, again, actin manipulation in both directions disrupted recruitment of RPA-2 to DSB foci. In nuclear HeLa extracts, RPA-3 had been previously suggested, but not validated, as a potential binding partner of actin by Serebryannyy et al.^37^. We now give experimental evidence that RPA-2 directly binds to nuclear actin in resting cells. Interestingly, Doxo-mediated DSB induction disassembled the nuclear RPA-2/actin interaction, most likely again by the induction of actin polymers. It could be speculated that RPA-2 is bound to a pool of nuclear G-actin in the absence of acute DNA damage, and that nuclear actin polymerization upon DSB induction is necessary to release RPA and to subsequently enable its recruitment to ssDNA. The observed inhibition of RPA-2 binding to actin by LB could be caused by allosteric effects of LB, which have previously been reported to inhibit binding of thymosin beta 4 to actin^38^. Interestingly, thymosin binds to the same region of actin^39^ which is suggested as binding site for RPA-2 in our docking model. Since we no longer see a recruitment of RPA-2 to DSBs after treatment with LB, we conclude that the existence of polymeric actin structures at the DSBs is mandatory for this process. For actin polymerizers we have recently shown a trapping of proteins in the resulting actin aggregates^30,35^. It could well be that RPA-2 shares a similar fate and is trapped in actin aggregates upon treatment with Jaspla, thus being no longer available for recruitment to DSBs. In these aggregates and at the DSB foci RPA-2 seems not to directly interact with actin, since these structures are negative in the proximity ligation assays. A graphic model of the suggested interactions of actin with RPA-2 and its disturbance by actin-binding compounds is depicted in Fig. 8. In therapeutical terms, inhibition of HDR is an attractive option because tumors with defects in HDR are often highly sensitive to DNA-damaging therapy^40^.

Interestingly, repair of DNA damage after etoposide treatment was not changed by actin-binding compounds. This could be due to the fact that the damage by etoposide is mainly mediated via inhibition of topoisomerase II, in contrast to the more complex damage caused by Doxo. Along this line, damage by irradiation, which is largely independent of topoisomerase II, was repaired to a lower degree in the presence of the actin binders. DNA damage caused by cisplatin is mainly repaired by nucleotide excision repair (NER)^41^. Since RPA-2 is also involved in NER^42^, our finding that cisplatin-induced damage is repaired to a lesser degree in the presence of actin binders underscores our hypothesis.

All in all, the simultaneous inactivation of different DSB repair mechanisms (NHEJ, HDR and SSA), as demonstrated in this work, makes actin binders promising tools for chemotherapy, as they target pathways that could otherwise substitute for each other. Most importantly, the observed DNA repair inhibition by application of actin-binding substances could also be achieved in vivo by tolerable, low doses of LB. So far, actin binders have not yet found their way into clinical therapy due to the fear of severe side effects. In our hands, DNA repair inhibition by actin-binding substances was achieved by concentrations below a cytotoxic threshold of the respective monotherapy. The issue of a rather narrow therapeutic window of actin-binding substances in vivo, as published for jasplakinolide^43^, might therefore not be a problem when actin binders are applied in low doses in a combination therapy setting like ours. In addition to our findings, previous reports on synergistic effects of actin-binding compounds with cytotoxic drugs^44,45^, e.g. by inhibition of efflux pumps make this approach even more promising.

As a consequence of this, we should bring actin binders back into focus for future (pre-) clinical studies as they do possess a high potential for the development of new combination treatment strategies in cancer.

## Supplementary information

Supplementary information
