# Supplementary material for: Targeting actin inhibits repair of doxorubicin-induced DNA damage: a novel therapeutic approach for combination therapy
Source: Cell Death Dis. 2019 Apr 3;10(4):302. doi: 10.1038/s41419-019-1546-9 (PMC6447524; doi:10.1038/s41419-019-1546-9)
Supplement: Supplementary file 1 — Supplementary information [file 41419_2019_1546_MOESM1_ESM.docx]

**Supplementary information**

**Materials and Methods**

**Antibodies**

The following antibodies were used: RPA-2 (ab2075, Abcam), actin (A2066/AMAB91241, Sigma Aldrich and MAB1501, Millipore), Ku70 (ab92450, Abcam), DNA-PKcs (12311, Cell Signaling Technology), yH2AX (2577, CST), p-ATM (5883, CST), p-Chk2 (2661, CST), topoisomerase II (D10G9, CST), HRP goat anti-rabbit (Bio-Rad, Munich, Germany), HRP goat anti-mouse (ab97240, Abcam), normal mouse IgG (sc2025, Santa Cruz), Alexa Fluor 488 (A11001, Thermo Fisher Scientific), PE (550083, BD Biosciences).

**Alkaline comet assay**

Cells were seeded in 0.7% low gelling agarose at 37°C on pre-coated glass slides (0.8% agarose) and lysed overnight (0.1M EDTA-Na_2_, 2.5M NaCl, 10mM Tris HCl, 1% Triton-X 100, NaOH, pH10). Electrophoresis was performed at 35V, 250mA for 15min, glass slides neutralized and DNA stained with GelRed™ (Biotium, Fermont, USA). Slides were fixed in 70% ethanol and sealed with FluorSave™ (EMD Millipore, Darmstadt, Germany). Images were taken with a Leica TCS SP8 SMD microscope (Leica Microsystem, Wetzlar, Germany) and analyzed with OpenComet (ImageJ).

**Duolink® assay**

Cells were fixed with 4% PFA, permeabilized wit 0.2% Triton X-100, blocked with 1% BSA in PBS and then incubated in primary antibodies. For substance treated samples, In Situ Detection Reagents Green were used, for YFP transfected cells In Situ Detection Reagents Red. Images were taken with a Leica TCS SP8 SMD and analyzed with ImageJ. Positive events in nuclei were counted manually.

**Nuclear run on assay**

To measure overall transcriptional activity, HeLa cells were incubated for 1h at 37°C with 5 mM 5-fluoro uracil (5-FU). After respective treatment of the cells, fluorescence was measured with a Leica TCS SP8 SMD microscope (Leica Microsystem, Wetzlar, Germany).

**Chromatin relaxation assay**

Fluorescence imaging was performed on a Zeiss AxioObserver Z1 inverted microscope with a spinning-disk scan head CSU-X1 from Yokogawa at a rotation speed of 5000 rpm and a Zeiss C-Apochromat 63x/1.2 water-immersion objective. Images were acquired on an AxioCam HRm CCD camera (Zeiss). A dedicated single-point scanning head (UGA-42 firefly from Rapp OptoElectronic) coupled to the epifluorescence backboard of the microscope was used for laser micro irradiation. Chromatin relaxation experiments were completed as previously described [1, 2]. Briefly, U2OS cells stably expressing PAGFP-H2B [3] were treated with the indicated actin binder or DMSO 90 min prior to imaging followed by Hoechst 33342 treatment (0.3 μg/mL) in combination with the actin binder or DMSO for 1 hour at 37°C. Immediately prior to imaging, growth media was replaced with CO_2_-independent imaging medium (Phenol Red-free Leibovitz’s L-15 medium (Life Technologies) supplemented with 20% fetal bovine serum, 2 mM glutamine, 100 µg/mL penicillin and 100 U/mL streptomycin) containing the actin binder or DMSO. Cells were irradiated with 405 nm light at a power density of 1 µJ/µm^2^ at the sample level. The laser power was measured and adjusted at the beginning of each experiment to ensure reproducibility. Cells were maintained at 37°C in the absence of CO_2_. Images were collected every 4 sec over a period of 2 min. The thickness of the photo activated line is measured over time using a customised Matlab routine and is displayed as a ratio of the thickness compared to the first time-point after irradiation.

**RPA-2/actin binding structure model**

The model of cytoplasmic actin was built using HHpred [4] using the structure of the mouse G-actin with 93% sequence identity as the template (PDB id 4B1Y). Protein-protein interaction interfaces of actin were predicted with PredUs [5] and SPPIDER [6]. Functionally important residues were predicted as highly conserved and exposed as identified by the ConSurf [7] web server. Structure for RPA-2 was obtained from the Protein Data Bank [8]. Global docking search was performed with ClusPro [9], and selected top-scoring poses were subjected to local docking with RosettaDock [10]. All methods were run with default parameters. Of 10 best global docking solutions produced by ClusPro, we selected those were the interacting partner was bound in the vicinity of this site, and where the energy profile showed the characteristic funnel-like shape, which is a sign of a good docking solution. They were further optimised with local docking tools RosettaDock, and the compact location of top-scoring docking poses was considered to be a further indication of a good docking result. Structures were visualized and in silico mutagenesis was performed with PyMol (Schrödinger, LLC).

**Western Blot and Co-immunoprecipitation**

Western blot lysis buffer: 2 mM EDTA, 137 mM NaCl, 10% glycerol, 2 mM Na_4_P_2_O_7_, 20 mM Tris-base, 1% Triton‑X 100, 20 mM sodiumglycerolphosphate, 10 mM NaF, 2 mM Na_3_VO_4_, 1 mM phenylmethylsulfonylfluoride and complete protease inhibitor (Sigma-Aldrich). Hypotonic lysis buffer: 10mM HEPES (pH7.9), 1.5mM MgCl_2_ 10mM KCL, 0.5mM DTT, 0.1% NP-40 (v/v): Nuclear lysis buffer: 20mM HEPES (pH7.9), 20% glycerol, 2mM MgCl_2_, 150mM KCL, 0.2mM EDTA, 0.5mM PMFS, 0.5mM DTT.

**Results**

**Effects of additional actin binding compounds**

Chivosazol A (an actin depolymerizer) and miuraenamide A (an actin polymerizer) both inhibit DNA repair after induction of DNA damage by Doxo (**Fig. S1**).


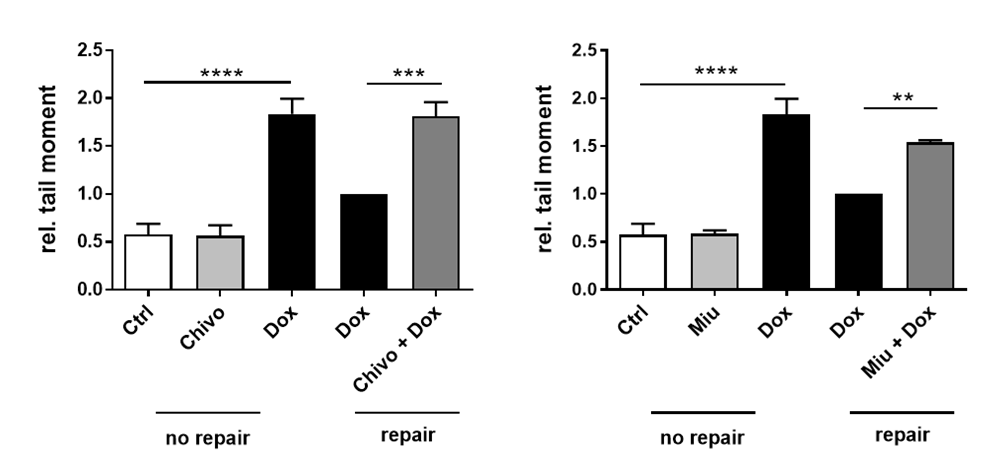


Figure S1

Actin manipulation inhibits repair of Doxo induced DNA damage. HeLa cells were treated with Doxo for 2h, medium removed and cells incubated in DMEM (repair time). Treatment without repair served as positive control, DMSO treatment as negative control. Alkaline comet assay was performed with cells treated additionally with the indicated actin substances. One-way ANOVA, **p<0.01, ***p<0.005, ****p<0.0001, n = 3.

**Nuclear actin visualization**

Pictures of all five cells of one round of FCS measurements are shown and fitting curves of one cell are depicted (**Fig. S2**)**.**


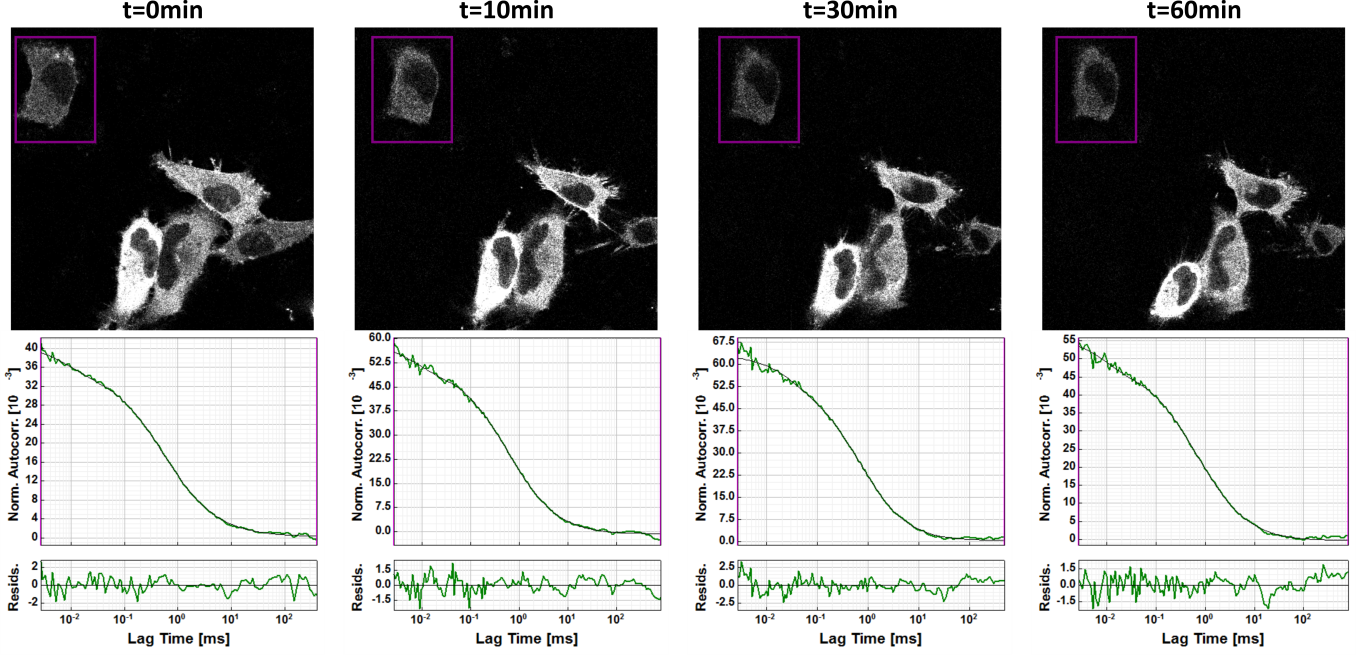


Figure S2

Example pictures of one round of single points FCS measurement (top) and fitted FCS autocorrelation curves and residual plots of one cell nucleus are depicted (bottom) .

**Effects of cell treatment on cell cycle and overall transcription**

Addition of actin binders did not as such cause a cell cycle arrest. Doxo caused a G2/M arrest, which was not altered by the co-treatment with the actin compounds (**Fig S3**, left panel). Overall transcription (incorporation of 5-FU) was also not influenced by the actin compounds. Doxo treatment caused a transient cessation od transcription, actinomycin D served as a positive control (**Fig. S3**, right panel).

Figure S3

Left panel: Influence of single and combination treatment with the compounds for 48h on cell cycle status of HeLa cells. LB and Jaspla have no effects, while Doxo causes a G2 arrest, which is not altered by combi treatment. Right panel: After DNA damage repair, none of the actin compounds affected transcription (incorporation of 5-FU), while actinomycein D completely blocked transcription. Transcription was blocked by Doxo treatment without repair time. One-way ANOVA, *p<0.05, n = 3.

**Effects of actin manipulation on early DSB signaling**

Addition of actin binders did not inhibit phosphorylation of ATM, Chk2 and H2AX (**Fig S4 A and B**) and chromatin relaxation was not impaired (**Fig S4 C**).


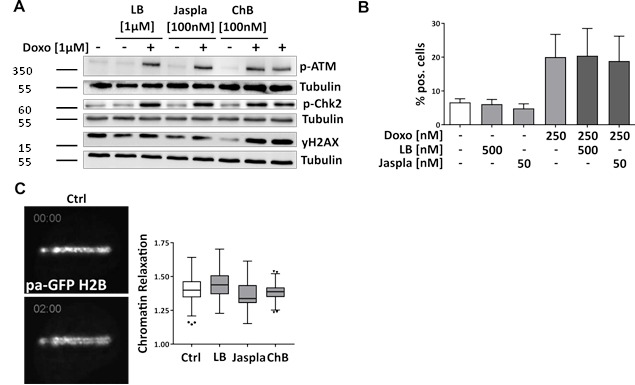


Figure S4

**A**, HeLa cells were treated with Doxo and the indicated substances for 1h. Cells were harvested, lysed and immunoblotting performed with antibodies against p-ATM, p-Chk2 and yH2AX. Tubulin served as loading control. One exemplary blot is shown out of three experiments. **B,** HeLa cells were treated with the indicated substances for 2h. Unbound protein was excluded by washing with extraction buffer, cells fixed with 4% PFA and stained for yH2AX and flow cytometry performed (n=3). **C,** U2OS pa-GFP H2B expressing cells were plated the day before. Prior to imaging, cells were pre-treated for 90 minutes with the indicated actin substances, followed by Hoechst treatment for 1h alone or in combination with the respective actin substance. Specific nuclear areas were photoactivated by the 405 nm laser to induce local DNA damage and chromatin decondensation was measured every 4 seconds for 120s (36 cells each sample, n=2-3). DMSO treated cells served as control (one exemplary image each for the time points at 0 and 2 minutes on the left).

Combination of actin binding compounds with other modes of DNA damage

Etoposide caused a pronounced DNA damage, which was quickly repaired. Latrunculin B or jasplakinolide had no influence (**Fig. S5**)

**
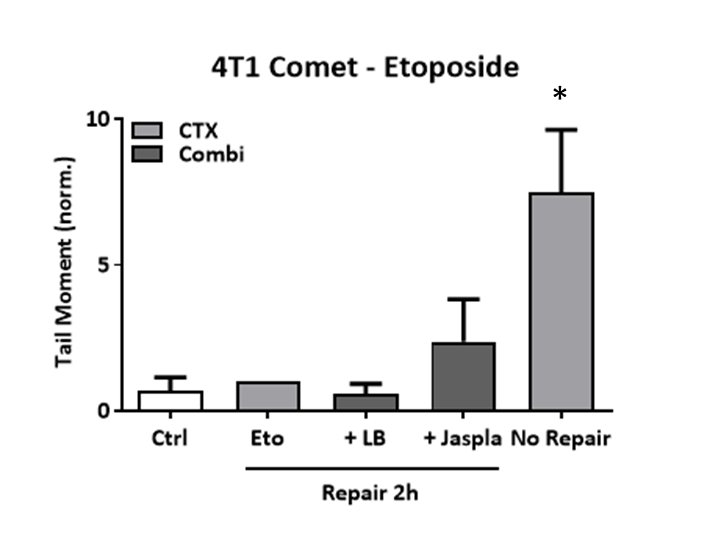
**

**Figure S5**

Actin binders do not inhibit repair of Eto (etoposide) induced DNA damage. 4T1 cells were treated with Eto for 2h, medium removed and cells incubated in DMEM (repair time). Treatment without repair served as positive control, DMSO treatment as negative control. Alkaline comet assay was performed with cells treated additionally with the indicated actin substances. One-way ANOVA, *p<0.05, n = 3.

Expression of topoisomerase II (topo II) was not changed by any of the actin compounds, Doxo or cisplatin (**Fig. S6**).


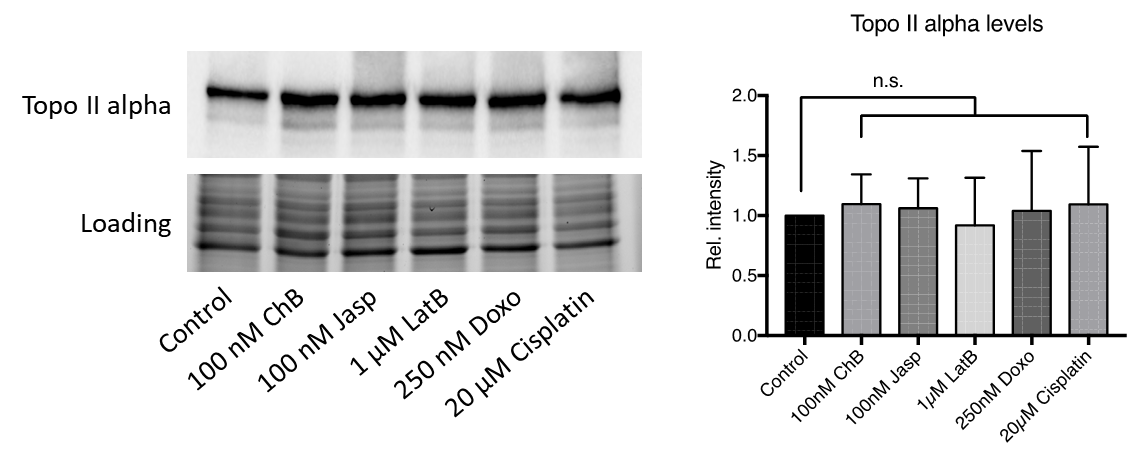

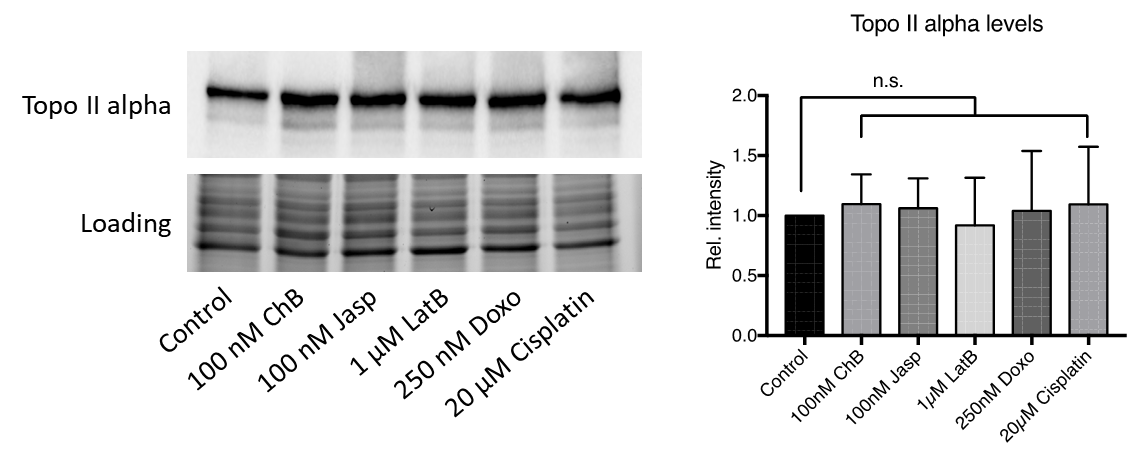


**Figure S6**

Neither chondramide B, nor jasplakinolide, latrunculin B, Doxo or cisplatin changed expression of topo II, as determined by Western blot (left upper panel). Left lower panel: loading control, right panel: densitometric quantification. One-way ANOVA, n.s. : not significant, n = 3.

Treatment of HeLa cells with cisplatin (20 µM) caused pronounced DNA damage (comet assay, **Fig. S7**, upper panel). This damage was quickly repaired. However, co-treatment with latrunculin B significantly inhibited this repair. The actin polymerizers chondramide B or jasplakinolide reduced repair in tendency, but without reaching statistic significance (comet assay, **Fig. S7**, upper panel). Cisplatin also led to a marked formation of RPA-2 foci (**Fig. S7**, lower panel). This effect was inhibited by both, jasplakinolide and latrunculin B (**Fig. S7**, lower panel).


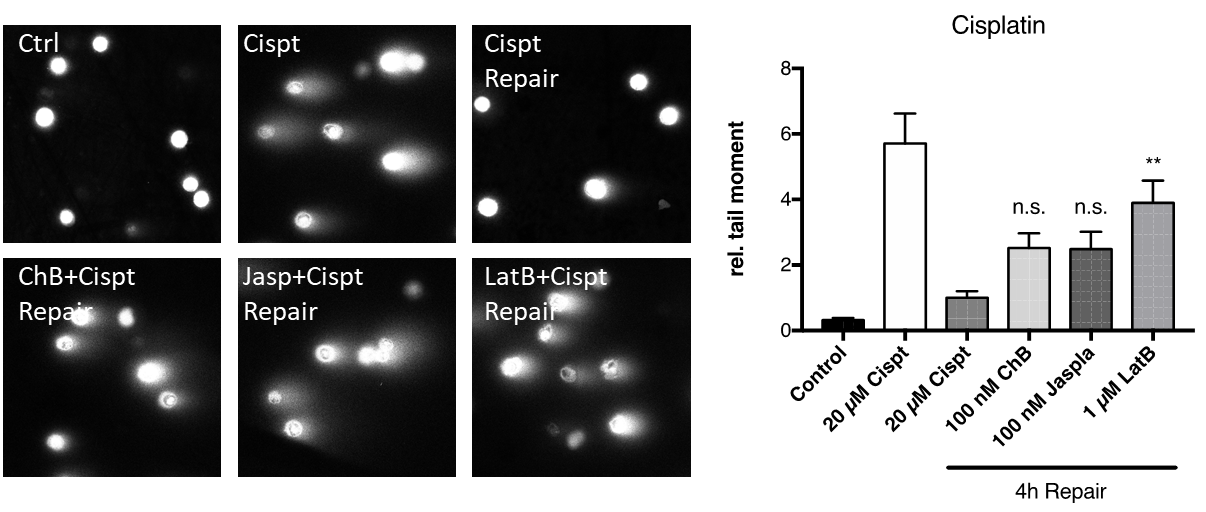


**
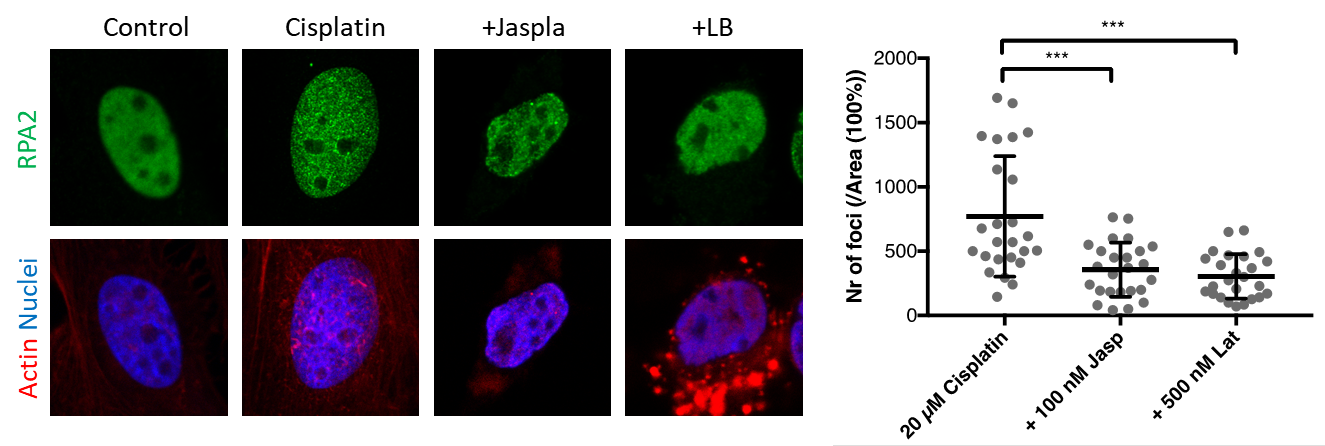
**

**Figure S7**

Upper panel: comet assay after treatment with cisplatin, 4h repair, and repair in combination with actin binding compouds. Left panel: representative images of cellular gel electrophoresis, right panel: quantitative evaluation of tail moment. Lower panel: RPA-2 foci formation after treatment with cisplatin, 4h repair, and repair in combination with actin binding compouds. Left panel: representative confocal images of cells stained with Hoechst (nuclei, blue), rhodamine phalloidin (actin, red) and a secondary Alexa488 antibody (RPA-2, green). One-way ANOVA, **p<0.01, ***p<0.005, n = 3.

Gamma irradiation of HeLa cells with an intensity of 2 Gy also caused pronounced DNA damage (comet assay, **Fig. S8**, upper panel). This damage was quickly repaired. However, co-treatment with chondramide B, jasplakinolide or latrunculin B significantly inhibited this repair (comet assay, **Fig. S8**, upper panel). Irradiation also led to a marked formation of RPA-2 foci (**Fig. S8**, lower panel). This effect was inhibited by both, jasplakinolide and latrunculin B (**Fig. S8**, lower panel).

**
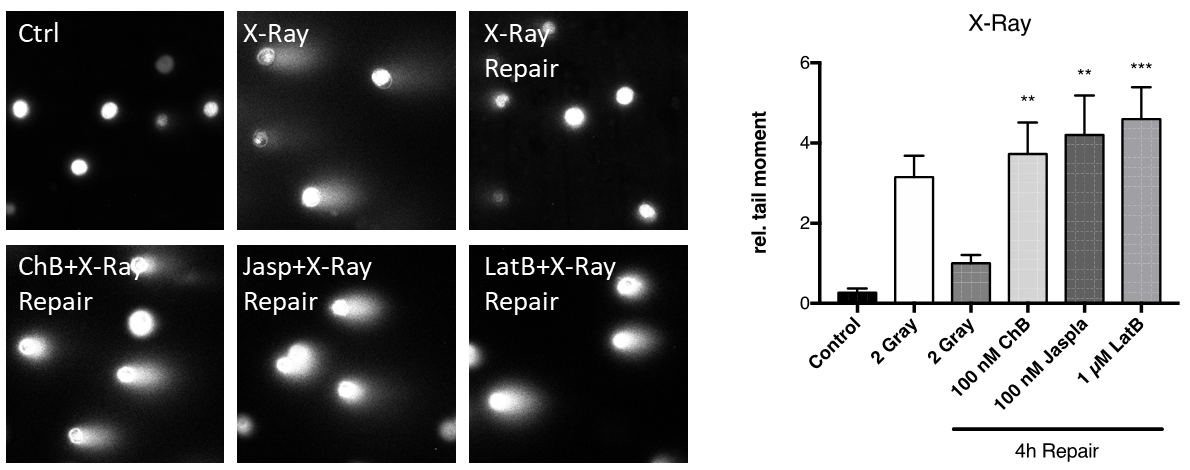
**

**
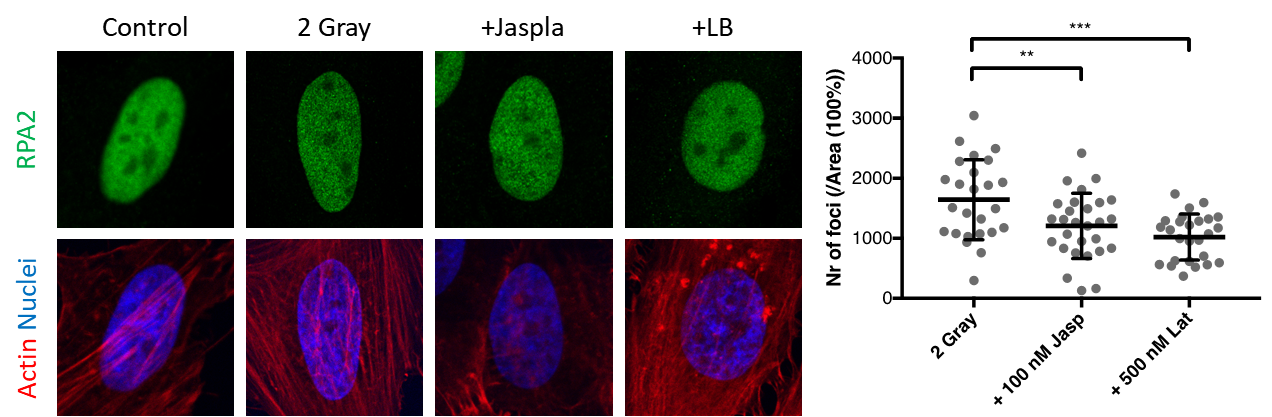
**

**Figure S8**

Upper panel: comet assay after treatment with an irradiation dose of 2Gy, 4h repair, and repair in combination with actin binding compouds. Left panel: representative images of cellular gel electrophoresis, right panel: quantitative evaluation of tail moment. Lower panel: RPA-2 foci formation after treatment with an irradiation dose of 2Gy, 4h repair, and repair in combination with actin binding compouds. Left panel: representative confocal images of cells stained with Hoechst (nuclei, blue), rhodamine phalloidin (actin, red) and a secondary Alexa488 antibody (RPA-2, green). One-way ANOVA, **p<0.01, ***p<0.005, n = 3.

**References**

[1] Sellou H, et al. The poly (ADP-ribose)-dependent chromatin remodeler Alc1 induces local chromatin relaxation upon DNA damage. Molecular biology of the cell. 2016;27(24):3791–3799.

[2] Lebeaupin T, Smith R, Huet S, Timinszky G. Poly (ADP-Ribose)-Dependent Chromatin Remodeling in DNA Repair. In: Poly (ADP-Ribose) Polymerase. Springer; 2017. p. 165–183.

[3] Smith R, Sellou H, Chapuis C, Huet S, Timinszky G. CHD3 and CHD4 recruitment and chromatin remodeling activity at DNA breaks is promoted by early poly (ADP-ribose)-dependent chromatin relaxation. Nucleic acids research. 2018;.

[4] Zimmermann L, et al. A Completely Reimplemented MPI Bioinformatics Toolkit with a New HHpred Server at its Core. Journal of molecular biology. 2017;.

[5] Zhang QC, Deng L, Fisher M, Guan J, Honig B, Petrey D. PredUs: a web server for predicting protein interfaces using structural neighbors. Nucleic acids research. 2011;39(suppl_2):W283–W287.

[6] Porollo A, Meller J. Prediction-based fingerprints of protein–protein interactions. Proteins: Structure, Function, and Bioinformatics. 2007;66(3):630–645.

[7] Ashkenazy H, Abadi S, Martz E, Chay O, Mayrose I, Pupko T, et al. ConSurf 2016: an improved methodology to estimate and visualize evolutionary conservation in macromolecules. Nucleic acids research. 2016;44(W1):W344–W350.

[8] Berman HM, et al. The protein data bank, 1999–. In: International Tables for Crystallography Volume F: Crystallography of biological macromolecules. Springer; 2006. p. 675–684.

[9] Kozakov D, et al. The ClusPro web server for protein–protein docking. Nature protocols. 2017;12(2):255.

[10] Lyskov S, Gray JJ. The RosettaDock server for local protein–protein docking. Nucleic acids research. 2008;36(suppl_2):W233–W238.
